# 1113. Ceftazidime retains *in vivo* efficacy against strains of *Stenotrophomonas maltophilia* for which traditional testing predicts resistance

**DOI:** 10.1093/ofid/ofad500.086

**Published:** 2023-11-27

**Authors:** Matthew C Phillips, Bosul Lee, Sarah Miller, Jun Yan, Marlene Maeusli, Rosemary She, Brian M Luna, Brad Spellberg

**Affiliations:** Massachusetts General Hospital, Cambrige, MA; University of Southern California, Los Angeles, California; University of Southern California, Los Angeles, California; University of Southern California, Los Angeles, California; University of Southern California, Los Angeles, California; Keck School of Medicine of the University of Southern California, Los Angeles, California; University of Southern California, Los Angeles, California; LAC+USC Medical Center, Los Angeles, California

## Abstract

**Background:**

*Stenotrophomonas maltophilia* is responsible for a growing number of nosocomial infections and is susceptible to a limited number of antibiotics. Recently the *in vivo* accuracy of conventional antibiotic resistance testing methods has been called into question. We sought to determine if there were efficacious antibiotics against *S maltophilia* that would be overlooked due to specious *in vivo* resistance determined by traditional methods.

**Methods:**

Antibiotic resistance testing was performed utilizing conventional and nutrient-limited media. Antibiotics with discordant minimum inhibitory concentrations (MICs) between the two media were selected for further experimentation. Metal ions were supplemented back into the nutrient-limited media to establish possible mechanisms. *In vivo* corroborations of *in vitro* MICs were done utilizing two infection models, *Galleria mellonella* and a neutropenic mouse oral aspiration pneumonia model.

**Results:**

*S. maltophilia* MICs were significantly lower for ceftazidime in nutritionally deficient than conventional media, resulting in a high percentage of strains determined resistant in traditional media being determined susceptible in nutritionally-deficient media. The addition of zinc and manganese to the deficient media abrogated this difference. Ceftazidime protected both *G. mellonella* and neutropenic mice against lethal infection caused by *S. maltophilia* that was predicted resistant in traditional media but sensitive in nutrient-deficient media.

S. Trophomonas more susceptible to ceftazidime (CAZ) but not aztreonam (ATM) and cefepime (FEP) in biologically relevant media

**Figure:**
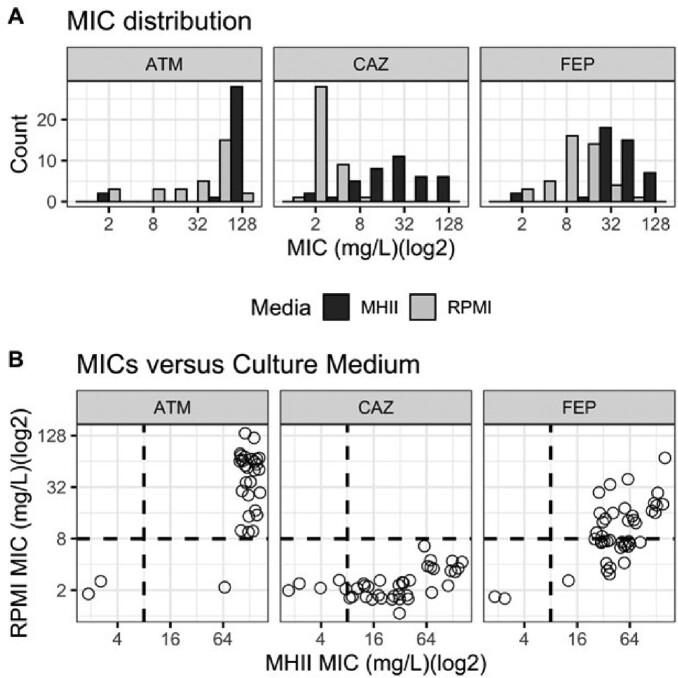

MICs were determined in MHII or RPMI-1640 media against an expanded panel of S. maltophilia clinical isolates. A) Comparing within drugs, there was a significant difference for MICs determined in MHII as compared to RPMI-1640 (p=6.89E-9 (ATM), 1.47E-11 (FEP), and 3.53E-13 (CAZ), Mann-Whitney). B) Axes represent different growth conditions. The dashed lines indicate the susceptible breakpoints. Everything to the left of the vertical line is predicted to be susceptible in MHII and everything below the horizontal line is predicted to be susceptible in RPMI-1640. Right lower quadrant represents isolates resistant in MHII but susceptible in RPMI-1640. CAZ was significantly different from ATM (Mann-Whitney, p=7.47E-6) and FEP (Mann-Whitney, p=7.41E-6). There was no significant difference between the ATM and FEP groups. All MICs determined for CAZ in RPMI-1640 were less than 8 mg/L and represent a susceptible breakpoint interpretation.

CAZ efficacy in a murine infection model

**Figure:**
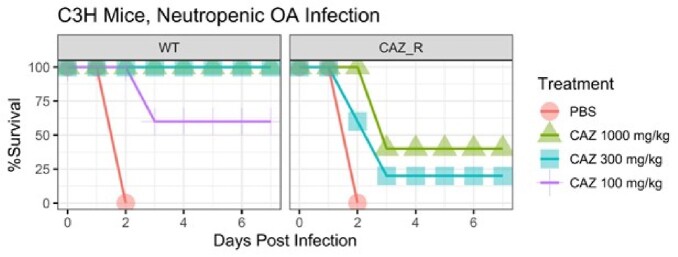

Dosing and dosing intervals for a human equivalent dosing strategy of 100 mg/kg were done as previously described. The dosing interval was kept constant, and doses administered were proportionally scaled up for the 300 mg/kg and 1000 mg/kg treatment groups.

**Conclusion:**

Ceftazidime may remain a viable therapeutic option for patients with *S. maltophilia* infection caused by strains predicted to be resistant by traditional susceptibility testing. Sequestration of trace metals in the host environment may prevent *S. maltophilia* beta-lactamase activity against ceftazidime.

**Disclosures:**

**All Authors**: No reported disclosures

